# Identification and Interpretation of Longitudinal Gene Expression Changes in Trauma

**DOI:** 10.1371/journal.pone.0014380

**Published:** 2010-12-20

**Authors:** Natasa Rajicic, Joseph Cuschieri, Dianne M. Finkelstein, Carol L. Miller-Graziano, Douglas Hayden, Lyle L. Moldawer, Ernest Moore, Grant O'Keefe, Kimberly Pelik, H. Shaw Warren, David A. Schoenfeld, and the Inflammation and the Host Response to Injury Large Scale Collaborative Research Program

**Affiliations:** 1 Pfizer Inc., New York, New York, United States of America; 2 Department of Surgery, University of Washington, Seattle, Washington, United States of America; 3 Biostatistics Center, Massachusetts General Hospital, Boston, Massachusetts, United States of America; 4 University of Rochester, Rochester, New York, United States of America; 5 University of Florida, Shands Hospital, Gainesville, Florida, United States of America; 6 University of Colorado, Denver Health Medical Center, Denver, Colorado, United States of America; 7 Division of Trauma and General Surgery, University of Washington, Harborview, Washington, United States of America; 8 Infectious Disease Unit, Massachusetts General Hospital, Charlestown, Massachusetts, United States of America; University of East Piedmont, Italy

## Abstract

**Rationale:**

The relationship between leukocyte gene expression and recovery of respiratory function after injury may provide information on the etiology of multiple organ dysfunction.

**Objectives:**

To find a list of genes for which expression after injury predicts respiratory recovery, and to identify which networks and pathways characterize these genes.

**Methods:**

Blood was sampled at 12 hours and at 1, 4, 7, 21 and 28 days from 147 patients who had been admitted to the hospital after blunt trauma. Leukocyte gene expression was measured using Affymetrix oligonucleotide arrays. A linear model, fit to each probe-set expression value, was used to impute the gene expression trajectory over the entire follow-up period. The proportional hazards model score test was used to calculate the statistical significance of each probe-set trajectory in predicting respiratory recovery. A list of genes was determined such that the expected proportion of false positive results was less than 10%. These genes were compared to the Gene Ontology for ‘response to stimulus’ and, using Ingenuity software, were mapped into networks and pathways.

**Measurements and Main Results:**

The median time to respiratory recovery was 6 days. There were 170 probe-sets representing 135 genes that were found to be related to respiratory recovery. These genes could be mapped to nine networks. Two known pathways that were activated were antigen processing and presentation and JAK- signaling.

**Conclusions:**

The examination of the relationship of gene expression over time with a patient's clinical course can provide information which may be useful in determining the mechanism of recovery or lack of recovery after severe injury.

## Introduction

There has been improvement in the outcome for acute lung injury (ALI) [1] but ALI and its sequelae multiple organ dysfunction syndrome (MODS) remain the leading cause of mortality after the first 24 hours post-injury [2]. These complications of trauma represent an enormous health care expenditure. Thus, the continued investigation of the pathogenesis of ALI/MODS remains a national research priority.

This paper is a preliminary report from the large scale collaboration Inflammation and the Host Response to Injury (*Glue*), a multi-centered study supported by the National Institute of General Medical Sciences (NIGMS), that aims to better describe genomic response in patients following severe injury or burns ([3], [4]). Our hypothesis was that we could find a list of genes for which expression levels predict a patient's clinical outcome, and that the identification of these genes could lead to new insights into the biology of MODS.

In a previous paper we showed that baseline gene expression could predict future MODS and other clinical events [5]. The predictor was a combination of thousands of gene expression values and thus could not provide insight into specific mechanisms.

Current thinking is that mechanical tissue disruption and cellular shock trigger a cascade of proinflammatory reactions, the systemic inflammatory response syndrome (SIRS). This primes the innate immune system such that a secondary insult during this vulnerable window provokes an unbridled inflammatory response culminating in early MODS [6]–[8]. The injury also initiates events resulting in a depressed adaptive immune response, counter inflammatory response syndrome (CARS), that renders the patient at risk for overwhelming infection, resulting in delayed MODS [9], [10].

We have investigated the mechanisms critical for early priming of the innate immune system and have employed the circulating PMN as a surrogate for this response [11], [12]. Our previous work has documented that injured patients at risk for MODS have a remarkably consistent pattern of post-injury PMN priming; beginning within 2 hr of injury, peaking at 6–12 hr, and resolving by 24 hr if there are no further insults [13]–[15].

Although the MODS is the primary mechanism of morbidity in the patients in this study there was often no clear period between the end of resuscitation and the onset of MODS. For this reason we choose respiratory recovery as a surrogate for MODS. In addition respiratory recovery represents a positive clinical outcome for a patient and can be viewed as a marker of an improving overall health.

In this paper, we apply novel statistical methods [17] to identify genes for which the expression trajectory predicts respiratory recovery, and then we relate these genes to the networks and pathways to which they belong.

## Materials and Methods

The analysis presented in this report includes data on a subset of 147 patients admitted to one of seven participating institutions, between November 2003 and July 2006. Our paper focuses on a subset of 147 patients who had complete genomic profiles at the time of our analysis. The *Glue* study entry criteria included patients who had suffered a blunt trauma without isolated head injury, who had arrived at a hospital within 6 hours of the injury, and had either hypotension or an elevated base deficit. Subjects with anticipated survival of less than 24 hours, significant pre-existing organ dysfunction, or severe traumatic brain injury were excluded. Written informed consent was obtained from all patients or their legally authorized representative. Blood was sampled at 12 hours and at 1, 4, 7, 21 and 28 days after the blunt trauma and was hybridized to an Affymetrix HU133 plus 2.0 gene chip. The details of the clinical protocol and sample processing are described in [5]. Respiratory recovery, the primary outcome of interest in this study, was defined as a patient's ability to breathe on their own after the removal of mechanical ventilation. The maximum follow-up time was 28 days, with patients who had not recovered by 28 days treated as censored with respect to the primary outcome at that time.

Gene expressions were extracted from oligonucleotide probes by a *perfect-match* model using dChip software (www.dChip.org) and gene expression values were log-transformed prior to any calculations. There were several steps taken to reduce the overwhelming dimensionality of the microarray. We first excluded probe-sets labeled ‘Absent’ over all arrays by the Affymetrix software. ‘Present/Absent’ labels in this technology indicate whether a probe-set was reliably detected or not. This step reduced the number to 48,992 probe-sets. Applying the assumption that the genes exhibiting temporal changes are potentially related to the outcome, we next excluded those probe-sets with a ‘low’ sample coefficient of variation, which we defined as having a coefficient of variation in the bottom half of the sample (i.e., below the sample median). This reduced the number of probe-sets to 11,461. Lastly, we performed the analysis of time-course microarrays using the EDGE [16] and deleted those that did not change with time. The number of investigated probe sets in the final dataset was 4,010.

The method used to obtain a list of statistically significant probe sets has been described previously [17]. The intention of this method is to apply a statistical test to assess whether the gene expression level predicts a subsequent event. In order to do this, we need the value of this expression level from each patient who is under observation and at “risk” for the event. Since this value will usually be missing, we need the approach described in [17] to handle the missing data. For this approach, we calculated one test statistic for each probe set among 4,010 by fitting a straight line to the expression level over time. We then used the estimated line to impute the probe set expression value at each time an event (respiratory recovery) occurred. We then calculated the difference between the imputed value for the patient who had the event and the mean of the imputed values for all patients who were on ventilator immediately before the event. This difference measures whether, at the event time, the observed expression level of the patient who had the event is greater or less than its expected value of the expression level if there were no relationship between the gene expression level and the occurrence of an event. The sum of these differences tests whether the expression levels are associated with the occurrence of an event.

Once we obtain a set of probe-set specific test statistics, we cannot use classical p-values to infer which probe-sets are important because the large number of hypothesis tests we have just done will result in an inflated type I error rate (inappropriate p-value). Instead, we can assess the statistical significance of each test by sorting them in descending order by their absolute value and decide how many genes in the list were statistically significant using a false positive ratio of 10% [18]. For this, we simulated the distribution of these statistics under the null hypothesis by permuting the event indicators among subjects at risk at that time. In other words, the number of subjects with events is kept fixed at each event time, but their event indicators are randomly exchanged among those currently at risk [17]. When we compared the simulated test statistics with our observed test statistics, we found that all but 170 probe-sets were eliminated, which preserved a false positive ratio of 10%. Thus we are accepting the fact that of the 170 probe-sets, 17 may be associated with a false positive test statistic [19].

## Results

**Figure 1:**
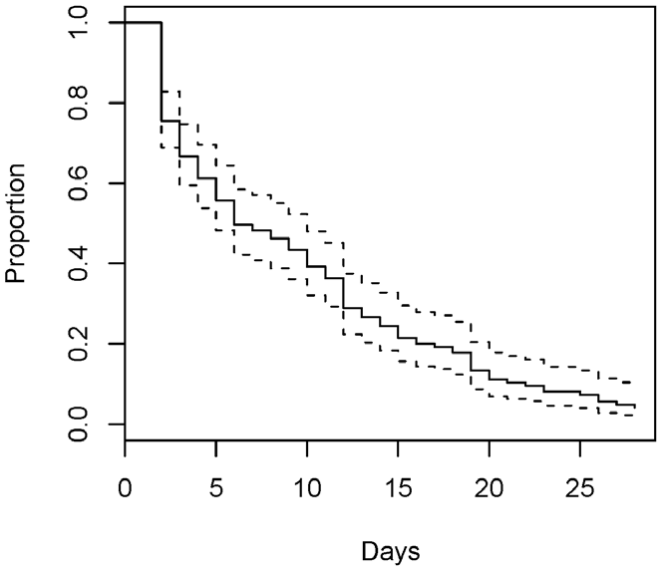
Kaplan-Meier plot of time to respiratory recovery.

Table 1 shows the demographic and clinical characteristics of the patients reported in this paper. Among 147 subjects, 11 did not experience respiratory recovery, including six patients who died during the study follow-up; for these 11 patients, time to recovery was censored. Figure 1 shows a Kaplan-Meier plot of the time to respiratory recovery. The median time to respiratory recovery was 6 days with a (2, 28) day range.

**Table 1:** Patient summary.

Median (range) or %	Total (n = 147)
Age	34 (16,55)
Gender, male	64%
Race	
White	88%
African Am.	6%
Asian	4%
Am. Indian	1%
Other	1%
Injury severity score	33 (6,75)
Max. MODS score	5.6 (0.4,16.4)
ICU days	10 (1,88)
ICU vent days	7 (0,48)

As described above, 4,010 probe-sets were evaluated for an association with the time to respiratory recovery. Of these, 170 were identified as statistically significant when the proportion of false positive findings is set at the 10% level. Using the WebGestalt [20] application, the 170 probe-sets were mapped onto 135 known genes. Of the 135 selected genes, 58 were found to be positively related to the time to respiratory recovery, while 77 genes were found to be negatively related to this event. Here, a positive association between a gene and time to a recovery means that the elevation in gene expression over the time prior to the event is associated with a shorter time to recovery. The opposite is true for the negatively associated genes, where elevation in gene expression prior to the event is associated with a prolonged time to respiratory recovery. Table 2 lists the subset of selected genes grouped by the direction of the association to the time to respiratory response. For example, elevated expression for IL12RB1 (interleukin 12 receptor) is associated with a shorter time to recovery.

**Table 2:** Description of a subset of selected genes found to be positively or negatively related to time to respiratory recovery.

**Elevation in expression is associated with shorter time to recovery**		
Symbol	Description	GO term (biological function)
APOL2	apolipoprotein L, 2	acute-phase response
CCR5	chemokine (C-C motif) receptor 5	inflammatory response
CD244	CD244 natural killer cell receptor 2B4	cellular defense response
CREBBP	CREB binding protein	signal transduction
DDX58	DEAD (Asp-Glu-Ala-Asp) box polypeptide 58	innate immune response
EGFR	epidermal growth factor receptor	MAP/ERK kinase activity
IL12RB1	interleukin 12 receptor, beta 1	antimicrobial humoral response
KIR2DL2	killer cell immunoglobulin-like receptor	immune response
NINJ1	ninjurin 1	cell adhesion
PSMB9	proteasome subunit, beta type	immune response
PSME1	proteasome activator subunit 1 (PA28 alpha)	immune response
SAMHD1	SAM domain and HD domain 1	immune response
TAP1	transporter 1	ATP binding
TAP2	transporter 2	ATP binding
**Elevation in expression is associated with longer time to recovery**		
Symbol	Description	GO term (biological function)
ALOX5AP	arachidonate 5-lipoxygenase-activating protein	inflammatory response
ANXA1	annexin A1	anti-apoptosis
DNAJC4	DnaJ (Hsp40) homolog	heat shock protein binding
HHEX	homeobox, hematopoietically expressed	antimicrobial humoral response
NR3C1	nuclear receptor subfamily 3	inflammatory response
PLP2	proteolipid protein 2	chemokine binding
POLI	polymerase (DNA directed) iota	DNA binding and repair
S100A12	S100 calcium binding protein A12	inflammatory response
S100A8	S100 calcium binding protein A8	inflammatory response

Legend: A subset of the 58 (77) genes found to be positively (negatively) related to the time to respiratory recovery is presented in the top (bottom) part of the table. For example, an elevated expression of APOL2 is associated with shorter time to recovery, while elevated expression of ALOX5AP is associated with a longer time to respiratory recovery.

**Figure 2:**
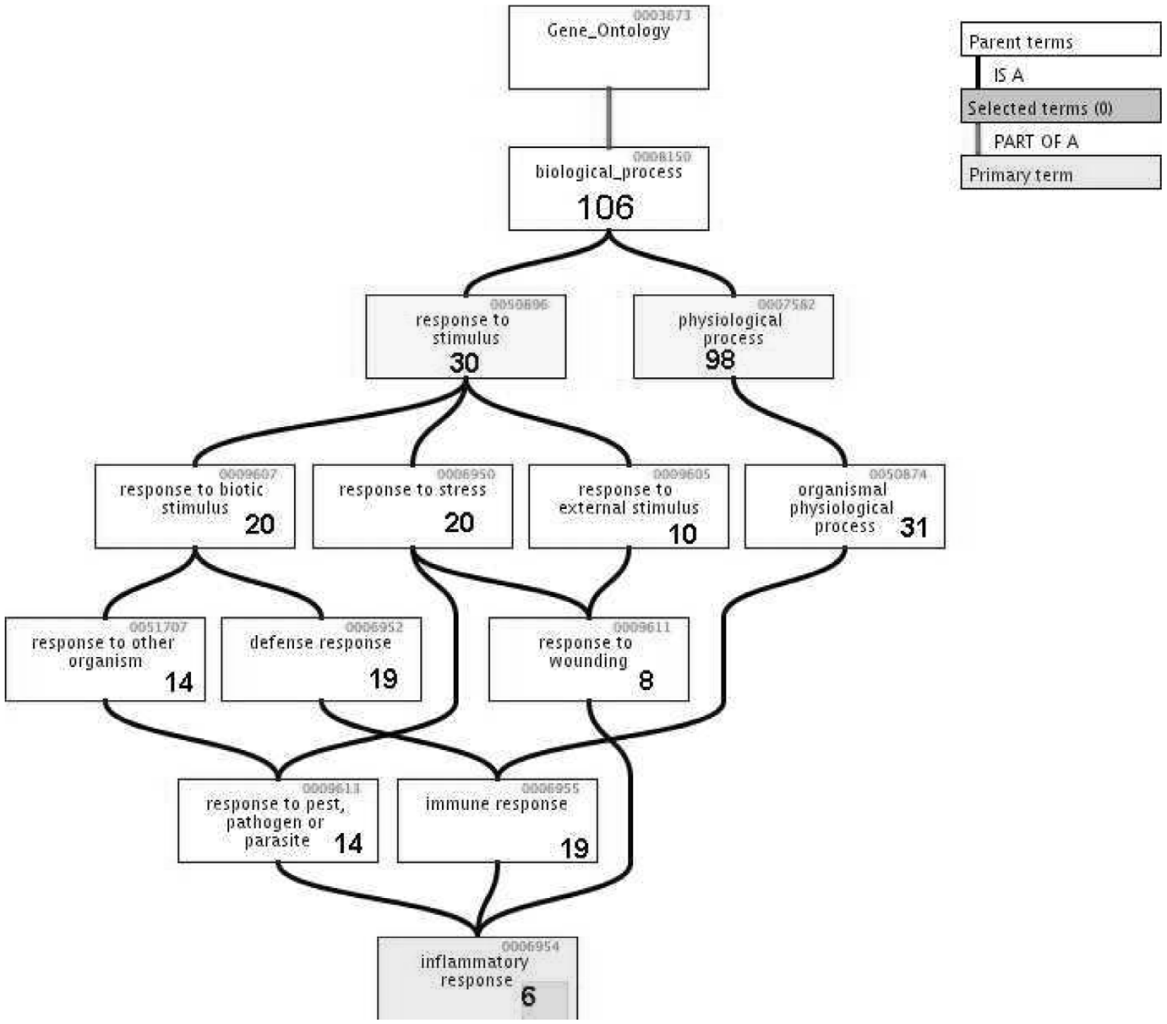
GO hierarchy of response.

We next examined the Gene Ontology (GO) terms corresponding to our group of genes found significant by statistical hypothesis testing. These are listed in Table 3. Figure 2 illustrates the hierarchy for the ontology terms comprising the *Response* branch of the biological process. Each node contains the number of genes among the identified genes in our set that are represented in that particular ontology term (this is a directed graph, where a line indicates precedence, so that the number of genes in a lower node is included in the total number of the preceding node). For example, a group of 30 genes were represented among the genes comprising *response to stimulus GO term*. The six genes that were represented in the *inflammatory response* GO term were ANXA1 (annexin A1), S100A8 (S100 calcium binding protein A8), ALOX5AP (arachidonate 5-lipoxygenase-activating protein), S100A12 (S100 calcium binding protein A12), CCR5 (chemokine (C-C motif) receptor 5), NR3C1 (glucocorticoid receptor).

**Table 3:** Six of the nine networks found by IPA.

Networks	Score	Focus	Functions
**ALOX5AP**, **APP**, APPBP2, **ATF1**, **C20ORF18**, **CCR5**, COL6A1	33	20	Gene Expression
**CREBBP**, **EGFR**, GLUL, GMEB1, GMEB2, **HHEX**, **HOXB2**			Tumor Morphology
HTR2A, **IL12RB1**, ITCH, **JAK2**, **LEPR**, **MACF1**, MGMT			Cancer
**MICAL1**, **NEDD9**, **NR3C1**, OSMR, **PIAS2**, **PML**, PTMS, RFP			
**RPS6KA3**, **SMAD3**, SNX4, SNX6, STAM2, TPSAB1			
ABCD2, **ANXA1**, **BCOR**, CASP1, CASP14, CEBPG	18	13	Lipid Metabolism
CLIC4, **DUSP6**, FABP1, FPRL1, HDAC9, HDAC1			Small Molecular Biochemistry
**HIVEP1**, ICEBERG, **IPO11**, **JARID2**, KLF5, MEFV			Molecular Transport
MNT, **NINJ1**, NUTF2, PCYT1A, **PEA15**, **PLP2**			
**PSMB9**, **PSME1**, PSME2, RAN, **SAMD4A**, SERPINB9			
**SET**, TGIF,TNF, UBE2E1, UBE2E3			
AKAP2, **AVO3**, **BET1L**, CAMKK1, CKAP2, CTPS	17	12	Cancer
**EPB41L2n**, EPB41L3, **FBXO9**, KLC3, MARK1			Tumor Morphology
**NCAM1**, NET1, **OAT**, PCTK2, **PRKAR2A**, PRODH			Cellular Development
RAPTOR, RNH1, RPS6KB1, SCOTIN, **SRGAP2**			
STARD10, STK11, SYNPO2, **TAP1**, **TAP2**, TGFA			
TGFB1, TP53, VDP, **WARS**, YWHAG, YWHAH, **ZNF175**			
**ARHGEF11**, C9ORF76, CASP3, CDKN2A, **CDV3**, DNAJB1	17	12	Cell Morphology
F2, GAST, GPX4, **GRHPR**, HD, HIP1, **HIP1R**, HNRPDL			Cancer
LAMP1, **MAP1LC3B**, MAPK14, MAPKAPK5, MCF2L, **NUDCD3**			Lipid Metabolism
**PRKD2**, RAB8A, RAC1, RPL7, RPS19, **S100A8**, S100A9			
**SH3GLB1**, TBX2, TCOF1, **TNS4**, TRIO, YBX2, **ZBTB10**, **ZNF385**			
AIF1, AKT3, ARHGDIA, **ASPH**, **BST1**, CD44, **CNP**	15	11	Cardiovascular System
CSRP1, **DDX58**, DTX1, **DTX3L**, EP400, GCH1, GGT1			Connective Tissue
**HLA-DMA**, IGF1, IGFBP6, INSR, NFIL3, **OSBPL7**			Organ Morphology
PA2G4, PCNA, PDCD4, PDE3B, **PGK1**, PHLDA1, POLD2			
POLH, **POLI**, PTEN, **SLC20A1**, **SYNCRIP**, TNFSF11, TRAIP, TUB			
CD48, **CD244**, CSTA, CTSC, CYP3A, **DMXL2**, EXPI	13	10	Cellular Function
FOS, **GAB2**, GCNT1, GRIN2B, GSTA1, IL2, IL3			Cell-To-Cell Signal
IL13, IL16, IL13RA2, INS1, KCNJ15, KIF17			Hematology System
**KIR2DL2**, LIN10, **LIN7A**, **LRP5**, PAEP, **PPM2C**			
**PPP1R12B**, PTPN22, RGD:632285, RPS7, RPS14			
**RPS4X**, **SNX3**, TCF1, TNFSF4			

Legend: Six of the nine genomic networks found by importing the 170 probe-sets into the Ingenuity Pathway Analysis (IPA) software. Focus genes (in bold) are genes from the input set represented in a particular network. Score of 3 or higher indicates that there is a 0.001 probability of a network being generated by chance alone.

**Figure 3:**
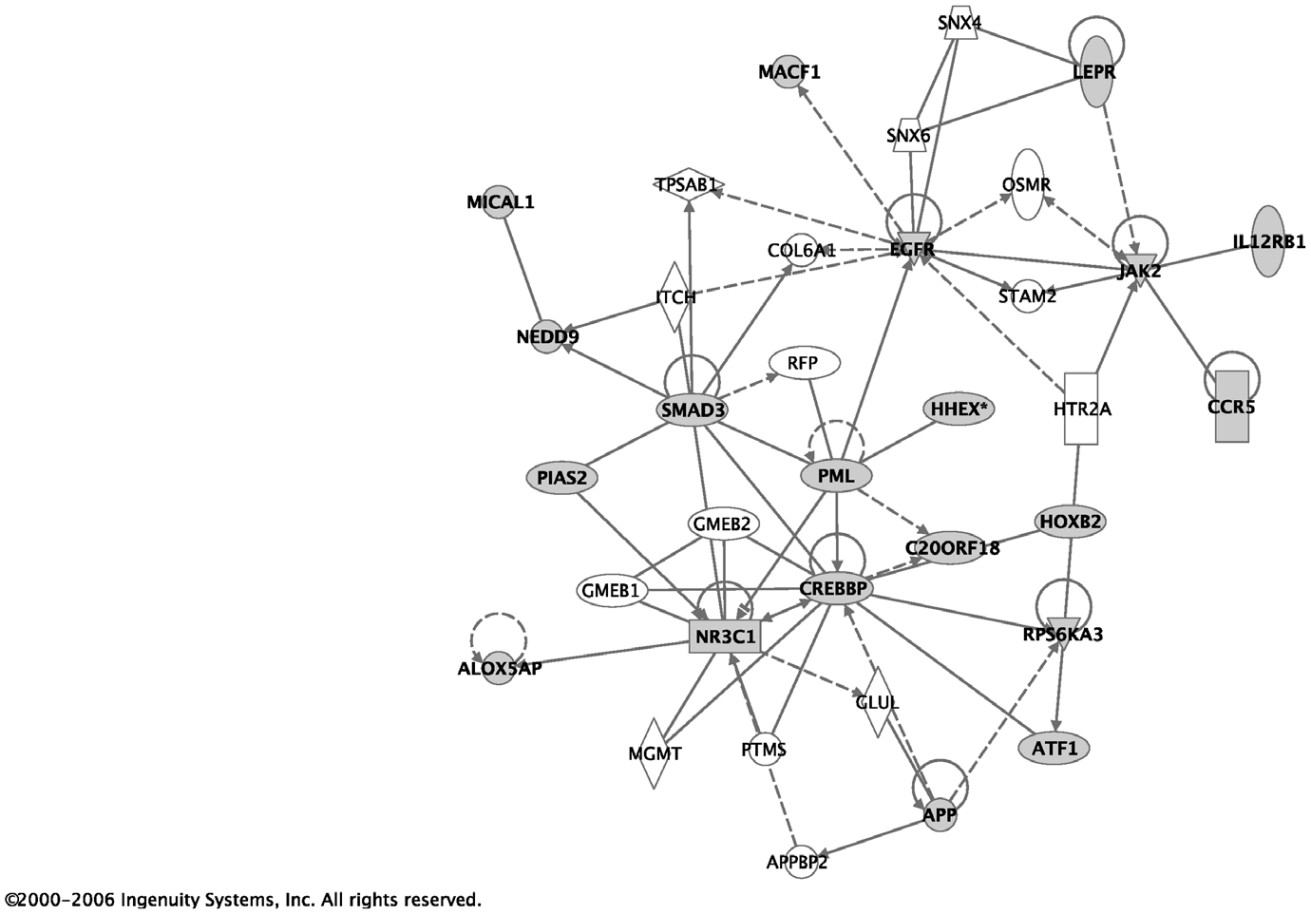
Network with the highest score. The network with the highest score in Table 3 is illustrated here. Shadowed boxes represent focus genes.

**Figure 4:**
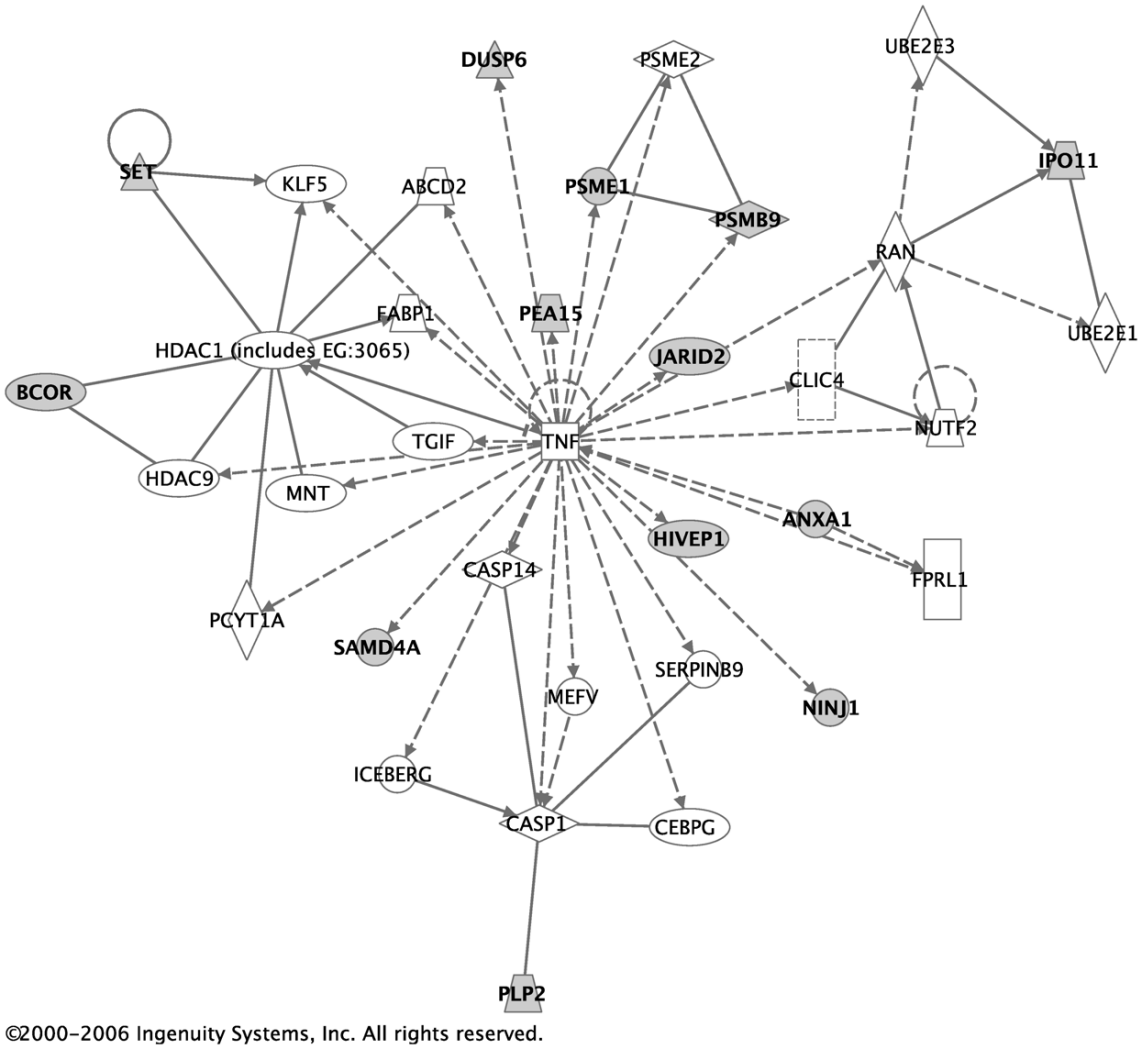
Network with the second-highest score. The network with the second-highest score in Table 3 is illustrated here. Shadowed boxes represent focus genes.

The 170 probe-sets with a false discovery rate (FDR) less than 0.1 were used in the network and pathway analysis. Affymetrix probe-set IDs were imported to the Ingenuity Pathway Analysis (IPA) software [21]. 168 of those probe-sets were mapped to the Ingenuity database. The identified genes were overlaid on the genomic network from the Ingenuity database and labeled as *Focus* genes. Connections for each focus gene were calculated by the percentage of its connections to other significant genes. A total of nine networks were found, six of which are shown in Table 3. For each network, all genes that compose it are listed. Focus genes are represented in bold. The remaining genes that appear in regular print were used to create each network and provide connections to the focus genes. For example, in the first network, there were 20 of the genes from our input set that were over-represented in that network relative to the size of the network. Each network is also assigned a score, which is a probability that the focus genes were represented in the particular network by chance alone. A network with a score of 3 or higher has a 0.001 probability of being generated by chance alone based on the Fisher's exact test. Figures 3 and 4 were created using Ingenuity software and represent the first two networks with the two highest scores. In these figures, shadowed boxes represent focus genes.

We next examined whether genes found in these networks can also be placed within known pathways. There exist large publicly available libraries of known networks and web application that allow for organized search and examination of these repositories. These web-based tools allowed us to examine further our list of selected genes and compare them to the configurations and content of known gene pathways. Pathways of highly interconnected genes were identified using the equation in [22].

For example, genes HLA-DMA, KIR2DL2, PSME1, TAP1, TAP2 were involved in the *antigen processing and presentation* pathway, while genes CREBBP, IL12RB1, JAK2, LEPR, MIZ1, ARCN2, CCR5, were involved in the *JAK-STAT signaling* pathway, and genes CREBBP, PLP2, and RPS6KA3 were in the Signaling Pathway from G-Protein Families.

## Discussion

Among the genes associated with a shorter time to recovery are IL-12RB and CREB that indicate IL-2 activation and CCR5 increase, the indicators of increased immune lymphocyte function. The Janus family (JAK-STAT) signaling pathway plays a critical role in signal transduction mediated by cytokine and hormone receptors. The JAK-STAT pathway is used by interferons and type I cytokines (cell products that that may stimulate immunity). These cytokines and interferons activate Janus family tyrosine kinases (JAK kinases), which in turn activate STAT proteins. In addition, JAKs are involved in the signal transduction pathways that govern cellular survival, proliferation, differentiation and apoptosis. It has been well documented in the literature that the loss of JAK kinase function has been found to result in disease states such as severe-combined immunodeficiency and that the optimal JAK kinase activity is crucial for normal cellular responses [23] of downstream signaling events. In support of this hypothesis, it has been found that JAK kinase function is required for optimal activation of the Src-kinase cascade, the Ras-MAP kinase pathway, the PI3K-AKT pathway and STAT signaling following the interaction of cytokine/interferon receptors with their ligands. Optimal JAK kinase activity is crucial for normal cellular responses.

Among the genes associated with a longer time to recovery is arachodoniate acid (ALOX5AP) that metabolizes into mediators of inflammation such as leukotrienes and prostaglandins and other ecocinoids which are associated with immune deactivation. Leukotrienes and prostaglandins act to increase vascular permeability and serves as chemoattractant for neutrophils. In addition, polymerase (DNA directed) iota (POLI) on the other hand has been reported to have enzymatic properties consistent with that of a somatic hypermutase and suggest that poliota may be one of the DNA polymerases hypothesized to participate in the hypermutation of immunoglobulin variable genes in vivo [24].

The major shortcoming of the current study is that it is based on a mixed cell population of leukocytes and genomic changes may reflect changes in this population. At the time of this study the technology was not available to obtain enough RNA from sorted cells to reliably run chips on pure cell populations. This technological hurdle has been overcome and we will soon be able to replicate this paper with pure cell populations.

Describing all the findings of a study such as this is difficult because the essential output of our analysis is a list of 170 probe sets included in the supplement. These probe sets were then mapped into 135 genes, and the genes where identified with networks and pathways that are also described in the supplement. We described these networks and pathways that are known to be associated with inflammation in order to validate the process that was used to find the 170 probe sets. This does not contribute new knowledge. New knowledge comes from the further study of some of the 135 genes that are not generally known to be related to inflammation or associations of our findings with other work that we are not aware of. We invite the reader to do this. A much larger data set with extensive genomic and clinical data is available by application on our website [3].
